# DC Surface Flashover Characteristics of Polyimide Containing Polyhedral Oligomeric Silsesquioxane (POSS) in the Main Chains under Vacuum

**DOI:** 10.3390/polym14122453

**Published:** 2022-06-16

**Authors:** Jian Wang, Ruofan Xiao, Renying Liu, An Ping, Zhe Wang, Jikui Liu, Shumin Zhang, Yanmin Liu

**Affiliations:** 1 State Key Laboratory of Alternate Electrical Power System with Renewable Energy Sources, Beijing 102206, China; 2 School of Electrical and Electronic Engineering, North China Electric Power University, Beijing 102206, China; x13938748081@163.com (R.X.); 120202201437@ncepu.edu.cn (R.L.); anping521794@163.com (A.P.); wz18810072775@163.com (Z.W.); 3 Beijing Institute of Control Engineering, Beijing 102206, China; sadabjljk@sina.com (J.L.); zhangsm15@tsinghua.org.cn (S.Z.); lym2020@foxmail.com (Y.L.)

**Keywords:** polyimide, POSS, DC surface flashover, vacuum

## Abstract

Polyimide, which is widely used to insulate power equipment operating in a vacuum environment, is prone to insulation failure due to surface flashover. Using POSS to modify it is an effective solution. This paper focuses on the study of DC surface flashover characteristics in vacuum of POSS/polyimide composite film, by introducing 1%, 3%, 5% equivalent mole content of POSS into polyimide, and conducting a surface flashover characteristics test in vacuum together with pure polyimide. The physical and chemical properties of the composite films were tested utilizing Fourier transform infrared spectroscopy and ultraviolet–visible spectroscopy. Combined with resistivity, SEM, and other test techniques, the influence mechanism of POSS molecular modification on DC surface flashover characteristics of polyimide films in vacuum was initially revealed. The results showed that after the introduction of POSS, the overall functional group structure of polyimide remained unchanged, the intermolecular charge transfer complexation was inhibited, and the transmittance of the film increased. The thermal conductivity and thermogravimetric temperature of the film are improved to a certain extent, and the mechanical properties are slightly decreased. With the increase of the introduced POSS content, the dielectric strength of the composite film is also enhanced. The surface flashover voltage of the composite film with a POSS content of 5% is 17.5 kV in vacuum, which is 30.5% higher than that of the pure film. Further analysis shows that the introduction of POSS will reduce the resistivity of the composite film, accelerate the dissipation of surface charges, and increase the flashover voltage. In addition, POSS forms a uniformly distributed Si-O-Si cage-like structure through molecular modification. When the surface of the film is damaged, SiOx inorganic flocculent particles are generated, which can not only scatter electrons, but also shallow the depth of trap energy level and accelerate the dissipation rate of surface charge, thus increasing the flashover voltage.

## 1. Introduction

Polyimide (PI) is a high-quality insulating material with excellent comprehensive properties. With its excellent electrical insulation, good radiation resistance, high mechanical strength, and excellent chemical resistance, it is widely used in power systems, low-temperature superconducting devices, and aerospace and other fields. When polyimide is used in power equipment, due to the influence of various factors, surface flashover may occur, resulting in power equipment failure. For example, the polyimide applied to the inter-turn insulation of power transformers is prone to surface flashover at the gas interface, which causes the failure of the gas-solid insulation structure of the power transformer [1]. In addition, for equipment operating in a vacuum environment, the failure caused by surface flashover of polyimide is also quite serious. In a low-temperature superconducting device, the superconducting magnet needs to be installed in the vacuum cavity to maintain the low-temperature environment in the liquid helium temperature region, so most of its insulating structures are in a high vacuum working environment. According to statistics, when the insulation system of the superconducting magnet fails, the polyimide as its insulating structure will have discharge phenomena such as surface flashover or even body breakdown, which is one of the main reasons for the failure of superconducting magnets [2]. When polyimide is used as an insulating material in a spacecraft, due to the combined effects of the rapid transformation and gradient distribution of high and low temperatures in space, high-energy charged particles, atomic oxygen [3], and the plasma environment, these external incentives will eventually lead to the phenomenon of surface flashover discharge in the aerospace environment of the polyimide insulating medium, and its insulating properties will be seriously damaged, which will lead to the failure of spacecraft components [4,5]. Therefore, to ensure the operational reliability of polyimide insulating materials in the fields of power systems, low-temperature superconducting devices, and aerospace, the academia and industry circles hope to improve the surface flashover characteristics of polyimide in vacuum through functional modification and other means.

At present, there are three methods to increase the flashover voltage of polyimide along the surface. The first effective strategy is to fluoridate the surface of the material to further enhance the surface flashover characteristics of the material under the premise of ensuring its excellent characteristics. Zhang [6] and others found that the flashover voltage of the polyimide sample after fluorination can reach 7.7 kV, which is twice the flashover voltage of the nonfluorinated sample. Liu [7] pointed out that the fluorinated film can produce a fluorinated layer with shallow trap depth, thereby inhibiting the accumulation of surface charges. However, the introduction of fluorine atoms in the process of polymer fluorination must be accompanied by corresponding structural changes, which will introduce corresponding physical and chemical defects in the surface layer [8]. Another approach is to dope polyimide with nanoparticles. Studies [9,10,11] have found that Al_2_O_3_, TiO_2_, SiO_2_, and other inorganic nanoparticles can significantly improve the electrical conductivity when they form a composite structure with polyimide, which accelerates the decay rate of space charges. In addition, these nanoparticles have ideal thermal conductivity, which can enhance the heat dissipation of polyimide, thereby reducing the injection of space charges, increasing the surface flashover voltage of polyimide. However, such methods also have their limitations. For example, the agglomeration of nanoparticles has always been a major problem in the modification of nanomaterials [12,13,14,15]. Another method is to carry out molecular modification of polyimide, introducing specific elements such as silicon and phosphorus into diamine monomer or dianhydride monomer, and introducing it into the molecular structure of polyimide through polymerization. Ding and Xing [16,17] used SiO_2_ nanoparticles and 1,3-bis(3-aminopropyl)-1,1,3,3-tetramethyldisiloxane (GAPD) to separate polyimide. It showed that both modification methods can obtain the better basic physical and chemical properties of polyimide such as thermal and mechanical properties. After adding 5% SiO_2_ and GAPD, the surface flashover voltage of the polyimide film was increased by 1.9 kV and 2.1 kV, respectively, and the surface discharge lifetime was increased to 3.4 times and 4.77 times that of the raw materials, respectively. It showed that the uniform distribution of the Si-O-Si into the polyimide will make the structure of the body more compact, and at the same time, the uniform flocculent non-polar particles will be formed after the outer layer is destroyed, thereby improving the creeping discharge characteristics of the polyimide. Polyhedral oligomeric silsesquioxane (POSS) has a Si-O-Si cage core [18], and its group has a high content of silicon, which can improve the surface flashover performance of the material, so it is an ideal molecular modifying group.

However, the current modification studies of POSS/polyimide composites mostly focus on the improvement of atomic oxygen resistance [19,20,21], and there is no relevant study on DC surface flashover characteristics of POSS/polyimide composites under vacuum. Therefore, a series of polyimide composite films containing POSS structures were prepared by in situ polymerization by using the idea of functional modification. In addition, in this study, the mechanical properties, electrical conductivity, thermal properties, mechanical properties, and vacuum DC surface flashover characteristics of the materials obtained with different POSS contents were studied in detail and compared with pure polyimide films of equal thickness.

## 2. Experimental Part

### 2.1. Materials

The diamine monomer is 4,4-diaminodiphenyl ether (ODA, 98%), the molecular formula is C_12_H_12_N_2_O, purchased from Shanghai Macklin Biochemical Co., Ltd. (Shanghai, China); the dianhydride monomer is pyromellitic anhydride (PMDA, 99%), the molecular formula is C_10_H_2_O_6_; purchased from Shanghai Macklin Biochemical Co., Ltd. (Shanghai, China); the solvent is *N*, *N*-Dimethylacetamide (DMAC, 99%), molecular formula is C_4_H_9_NO, purchased from Shanghai Macklin Biochemical Co., Ltd. (Shanghai, China); the modified material is amine-functionalized polyhedral oligomeric silsesquioxane (POSS, 95%), the molecular formula is C_48_H_48_N_8_O_12_Si_8_, purchased from Shanghai Macklin Biochemical Co., Ltd. (Shanghai, China) All chemicals and materials were used as received.

### 2.2. Preparaion of Polymers

**Figure 1:**
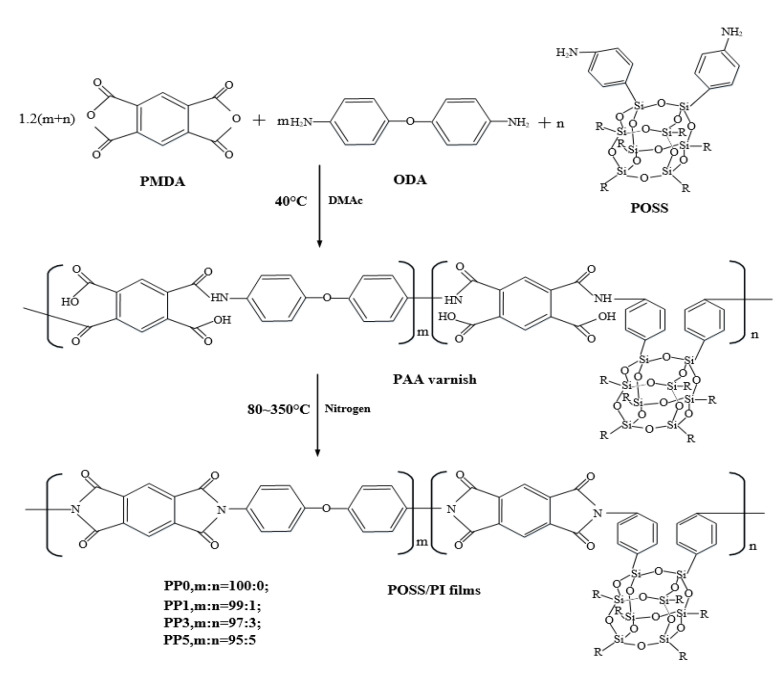
Synthesis route of POSS/PI composite film.

The modified polyimide composite film containing POSS was prepared by using a relatively mature two-step method in the laboratory. Considering that PMDA inevitably undergoes hydrolysis during preparation, it is best to have a slight excess of PMDA. According to the preparation experience in our laboratory, when the molar content ratio of PMDA and ODA is 1.02:1, the molecular mass of the prepared product is the most ideal. Therefore, we used 15.3 mmol PMDA and 15 mmol ODA to prepare polyimide films and based on the total molar content (15 mmol), added 1%, 3%, and 5% molar content of POSS to polyimide molecules. The specific modification scheme is shown in Table 1. The pure polyimide film was named PP0, and the composite films were named PP1, PP3, and PP5 according to the content of POSS. The synthetic route of the POSS/polyimide composite film is shown in Figure 1.

**Figure 2:**
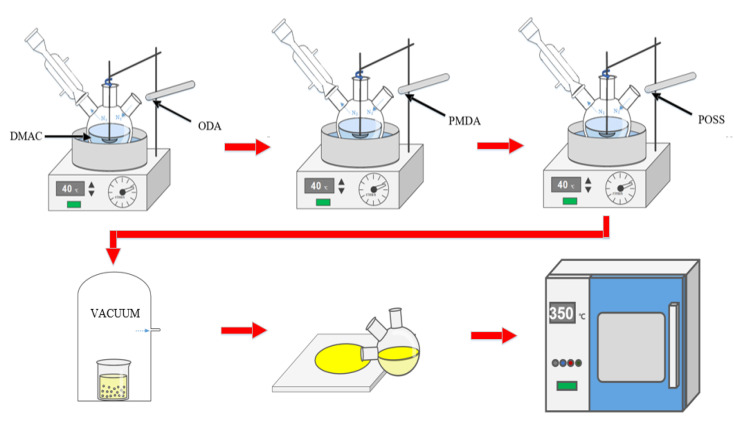
Preparation process of polyimide composite film.

The film preparation process is shown in Figure 2. In a three-necked flask (150 mL) equipped with an electromagnetic stirring device and a nitrogen protection device, 15 mmol of ODA was added and the flask was placed in a nitrogen atmosphere. It was then mechanically stirred for 2 h in a 40 °C oil bath to be fully dissolved in 35 mL DMAc solvent. Subsequently, the PMDA required for each scheme was added three times at a ratio of 6:3:1, the bottle wall was rinsed with a small amount of solvent after each addition, and the time interval for each addition was 0.5 h. Mechanical stirring was carried out for 6 h in a nitrogen environment, and then the specified content of POSS nanofillers (1%, 3%, 5%) was added, and a viscous PAA/POSS composite solution was obtained after mechanical stirring for 6 h again.

After the solution obtained from the above reaction was subjected to 0.5 h defoaming treatment (vacuum pumping), the solution was evenly coated on the glass sheet that was cleaned by ultrasonic cleaning; then it was transferred to a high-temperature oven for gradient heating (the heating process was 60° C × 1 h, 80 °C × 1 h, 150 °C × 1 h, 180 °C × 1 h, 210 °C × 0.5 h, 240 °C × 0.5 h, 270 °C × 0.5 h, 300 °C × 0.5 h, 350 °C × 0.5 h); finally, the film-containing glass plate that was naturally cooled to room temperature was placed in deionized water for 0.5 h, and the polyimide nanocomposite film was obtained after defilming, with a thickness of 40 ± 4 μm.

### 2.3. Characterization

In this study, the micro molecular structure of polyimide nanocomposite film samples was obtained by infrared spectroscopy to characterize the integrity of the molecular structure modification and thermal imide preparation. It was determined by ThermoFisher Technology Niclet IS50 Fourier transform infrared spectrometer (Waltham, MA, USA), and the band scanning range was 4000~400 cm^−1^. The samples were tested for structural characterization and charge transfer complexation (CTC) using a Shimadzu (Suzhou, China) (UV3600) ultraviolet–visible spectrophotometer (UV–Vis). The surface morphology of polyimide composite film before and after flashover were observed by JSM-6700F cold field emission scanning electron microscope from JEOL Ltd (Beijing, China). To test the thermal properties of the modified polyimide, it was analyzed using a high-temperature TGA/DSC synchronous thermal analyzer of the Swiss Mettler Toledo SDT Q600. During the test, it was protected by N_2_, the heating rate was 10 °C/min, and the temperature range was 20 °C~800 °C.

To characterize the resistivity of the material and its effect on the surface flashover in vacuum, the volume resistivity and surface resistivity of the polyimide nanocomposite thin film samples were measured by the Keithley 6517B high resistance meter and the 8009 three-electrode fixture. To characterize the thermal conductivity of the modified material, the thermal conductivity of the polyimide nanocomposite film samples was tested by Xiangtan Xiangyi DRL-III thermal conductivity tester (Changsha, China). To characterize the mechanical properties of the modified materials, the tensile strength and other properties of the polyimide nanocomposite film samples were tested by a high-speed iron testing instrument A1-7000M tensile testing machine (Shanghai, China).

**Figure 3:**
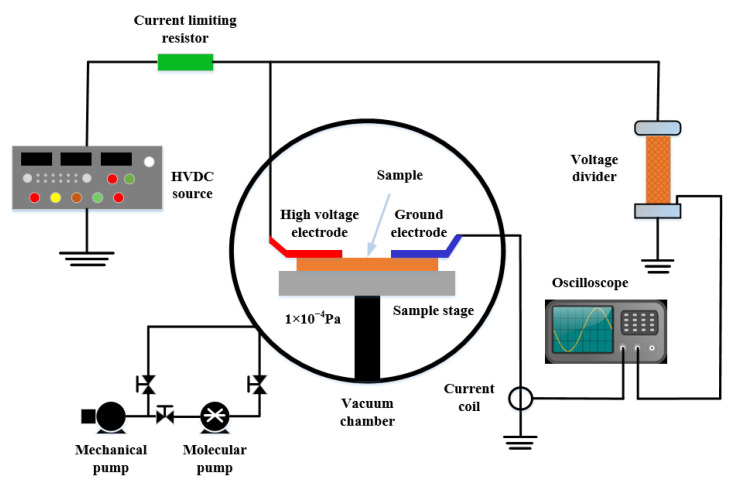
Vacuum surface flashover platform.

The vacuum surface flashover experimental platform was independently built by the research group. The schematic diagram of the platform is shown in Figure 3. The size of the vacuum chamber is 500 mm × 500 mm × 500 mm, the vacuum degree inside the chamber is 1 × 10^5^ Pa ~1 × 10^−4^ Pa when the equipment is running, the control environment temperature is 293K, and the relative humidity is 30%. A DC electric field was established using finger electrodes with a length of 40 mm, a width of 15 mm, and a height of 5 mm. A DC high voltage source (output voltage 0~35 kV) was used to provide a DC high voltage to the electrode through a 100 MΩ protection resistor. The size of the sample used in the experiment was 50 mm × 40 mm × 0.04 mm. Before the experiment, the sample was cleaned with absolute ethanol and placed in an oven at 60 °C for 24 h. Before the experiment, the distance between the finger electrodes was set to 3 mm, the insulating lifting platform in the cavity was used to make the polyimide composite film close to the lower end of the finger electrodes, and an epoxy resin insulating sheet was placed under the film. The DC high voltage source is connected to the surface flashover experimental platform in the vacuum chamber through the casing and then grounded through the aviation plug inside the chamber. During the experiment, the voltage was continuously boosted at a boosting rate of 0.1 kV/s until the film was powered off when surface flashover occurred, and then the amplitude of the flashover voltage was recorded, and a high-speed camera (Fastec Hi Spec5, Beyond Optoelectronics Technology Co., Ltd., Shanghai, China) was used to record the flashover morphology.

## 3. Experimental Results and Discussion

### 3.1. Structural Characterization

**Figure 4:**
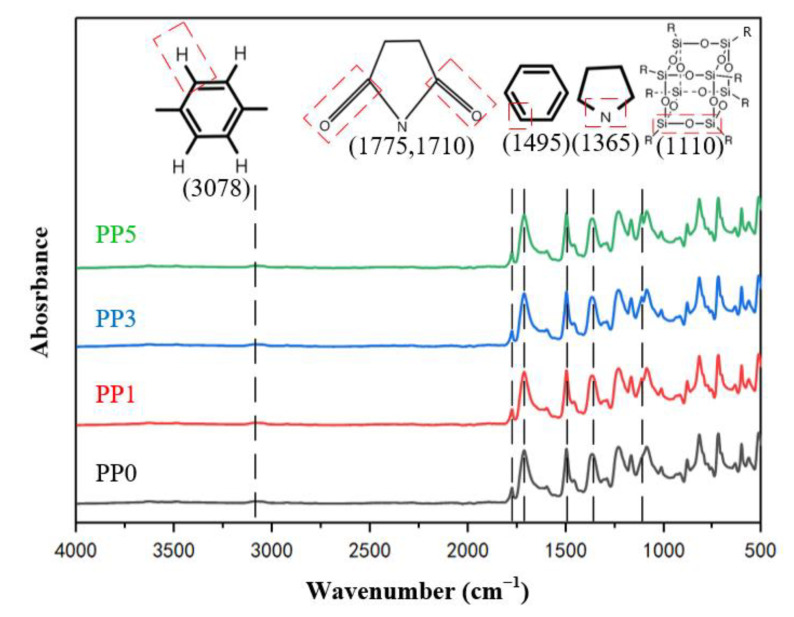
Infrared absorption spectrum of films.

The molecular structure properties of composite films with different POSS contents were characterized by infrared spectroscopy. Figure 4 shows the ATR-FTIR spectrum of the film along with representative absorption peaks, and some representative functional groups are indicated. There are asymmetric carbonyl (C=O) stretching vibration absorption peaks at 1775 cm^−1^, symmetric carbonyl (C=O) stretching vibration absorption peaks at 1710 cm^−1^, and carbonyl (C=O) bending vibration absorption peaks at 722 cm^−1^ in each sample. There is a stretching vibration absorption peak of C-N group at 1365 cm^−1^, indicating that all samples have formed imide five-membered ring.

There is no stretching vibration absorption peak of carboxyl group (-OH) near 3200 cm^−1^, no amino N-H stretching vibration absorption peak at 3350 cm^−1^, and no absorption peak of -CONH group at 1700 cm^−1^, showing that the dehydration condensation reaction of CONH and COOH in polyamide acid was complete, so all samples completed thermal imidization through multi-stage heating. At 1495 cm^−1^, there is a stretch characteristic absorption peak representing the C-C bond in the benzene ring, and at 3078 cm^−1^, there is a stretch characteristic absorption peak representing the C-H bond in the benzene ring, indicating that the aromatic structure of the film is complete. At 1110 cm^−1^, PP0 has no characteristic peak, and the stretching vibration of Si-O-Si can be observed at 1110 cm^−1^ of the PP1, PP3, and PP5 curves, indicating the successful introduction of POSS in the PI film [22].

### 3.2. Optical Properties

**Figure 5:**
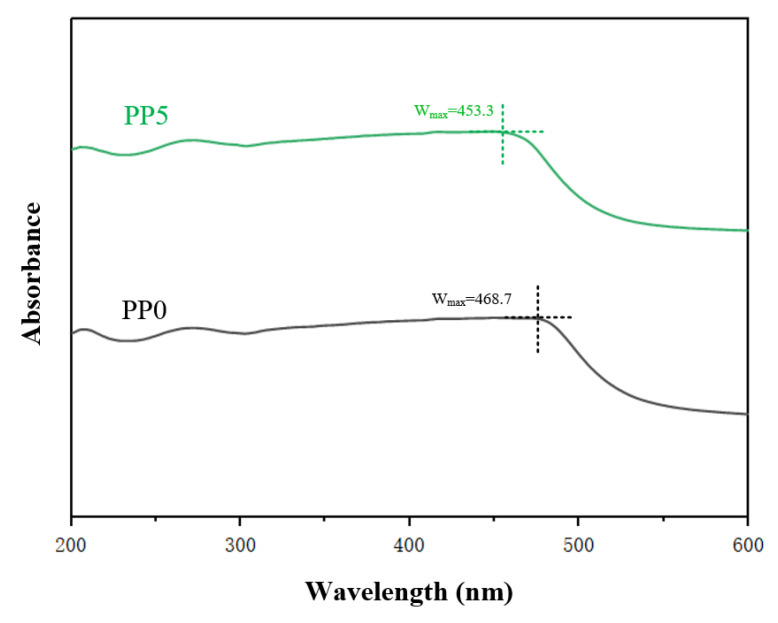
Ultraviolet visible absorption spectrum of films.

Polyimide is composed of diamine and dianhydride, the former and the latter are electron donor and electron acceptor, and the packing of polyimide molecules is affected by the charge transfer complexation between electron donor (diamine chain) and electron acceptor (dianhydride chain). The structure characteristics and charge transfer complexation of polyimide films with different POSS contents were characterized by ultraviolet spectroscopy. The 200~600 nm UV–Vis spectrum is shown in Figure 5.

The wavelength at the peak in the ultraviolet spectrum curve is defined as the maximum absorption wavelength W_max_, and the maximum absorption wavelength shift trend of PP0 and PP5 is analyzed based on this. It can be seen from Figure 5 that after the introduction of POSS, the W_max_ of the near-ultraviolet region and the visible light region moves to the short-wave direction, that is, a blue-shift phenomenon occurs. The reason for this phenomenon is that POSS is introduced into the polyimide molecular chain by chemical bonding (Figure 1), and POSS replaces the original diamine structure. After the introduction of POSS, the ability of the material to absorb long waves is inhibited. It reduced the formation of charge transfer complexes between polyimide molecular chains and reduced the conjugated structure of the polymer system, thus weakening the intermolecular force. In addition, according to the data, the peak at the wavelength of maximum absorption of the thin film material decreases with the increase of POSS content. This is mainly attributable to the fact that the cage-like structure of POSS fillers weakens the packing density of polyimide molecular chains, thus facilitating the transmission of visible light.

### 3.3. Thermal and Mechanical Properties

**Figure 6:**
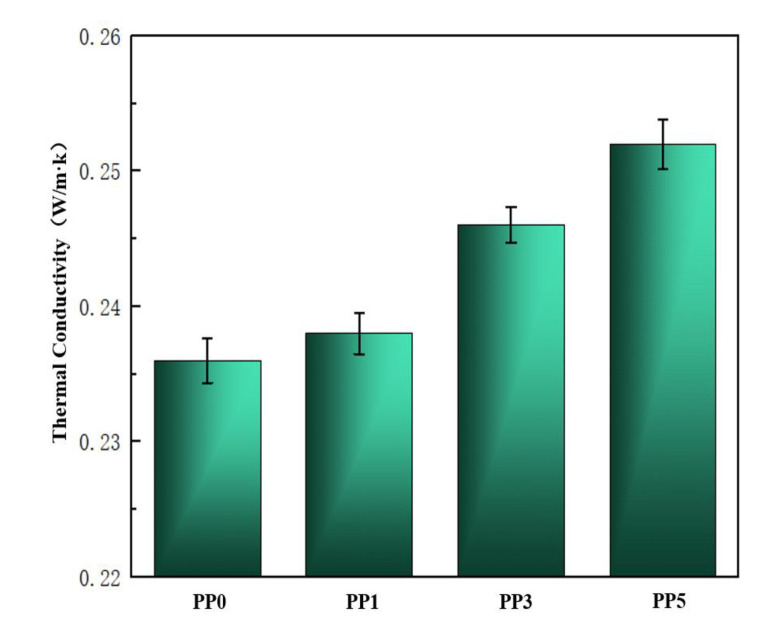
Thermal conductivity of films.

Thermogravimetric analysis (TGA) and thermal conductivity λ curves were performed on the samples to study the effect of POSS units on the thermal properties of PI films. The thermal performance data are shown in Table 2, and the thermal conductivity curve is shown in Figure 6. It is very difficult to dissipate heat in a vacuum environment, so the heat dissipation capability of the material is very important. Polyimide has good high-temperature resistance, but its thermal conductivity is low and its heat dissipation capacity is poor. As shown in Figure 6, with the increase of POSS content, the thermal conductivity of the composite film will increase slightly. When the POSS content is 5%, its thermal conductivity is 0.252 W/m·k, which is 7.1% higher than that of the pure film.

As shown in Table 2, the thermal and mechanical properties of the films were tested five times and averaged, and the sample standard deviation was recorded after the data. In this paper, the thermal stability of polyimide composite films in nitrogen atmosphere was analyzed by TGA tester. According to IEC-60216-2-2005, if the mass loss of the material exceeds 5%, the electrical insulation performance of the material is judged to fail. Therefore, in this study, the T_5%_ thermogravimetric loss temperature of each sample was calculated, and the percentage value of the remaining mass of the material after the test at 750 °C was recorded. As shown in Table 2, the T_5%_ of pure polyimide is at 532 °C, and with the increase of POSS content, the temperature at the sample T_5%_ also increases. The T_5%_ of PP5 can reach 552 °C. The reason is that the phenyl and cubic silicon cores with good thermal stability and the stable covalent bonds between the components limit the continuous decomposition of the polyimide phase. In addition, the R_W750_ of the samples also increased with the increase of the POSS content, which was due to the oxidation reaction of silicon element with a trace amount of oxygen in the gas at high temperature, resulting in the formation of heat-resistant silicon oxide [23].

In practical applications, the tensile properties of the film are a very important factor in judging its reliability, especially when the film is used in aerospace and other difficult-to-repair environments. In these application environments, films with good tensile strength, high tensile modulus, and excellent elongation at break are often required. From the data in Table 2, it can be analyzed that the tensile strength and elongation at break of the composite film decrease with the increase of the POSS content in the polymer. The tensile strength of the sample with 5% POSS content is 107.6 MPa, which is 21.1% lower than that of pure polyimide, and the elongation at break is 14.9% lower. This is mainly because the steric hindrance effect of POSS is more obvious due to its unique cage structure, and compared with ODA, POSS-diamine may have lower reactivity, which eventually leads to the reduction of the molecular weight of the composite film and the inefficient chain–chain stacking. After the introduction of POSS into polyimide, the rigidity of the molecular chain is enhanced, and the fracture mechanism of the composite film changes from toughness to brittleness [24], so its elongation at break gradually decreases. However, when the POSS content is 5%, the elongation at break of the composite film is still 30.3%, indicating that the polymer chain still exhibits certain molecular flexibility. In general, the polyimide composite film with 5% POSS content can still meet the requirements of most of its application scenarios.

### 3.4. Surface Flashover in Vacuum

**Figure 7:**
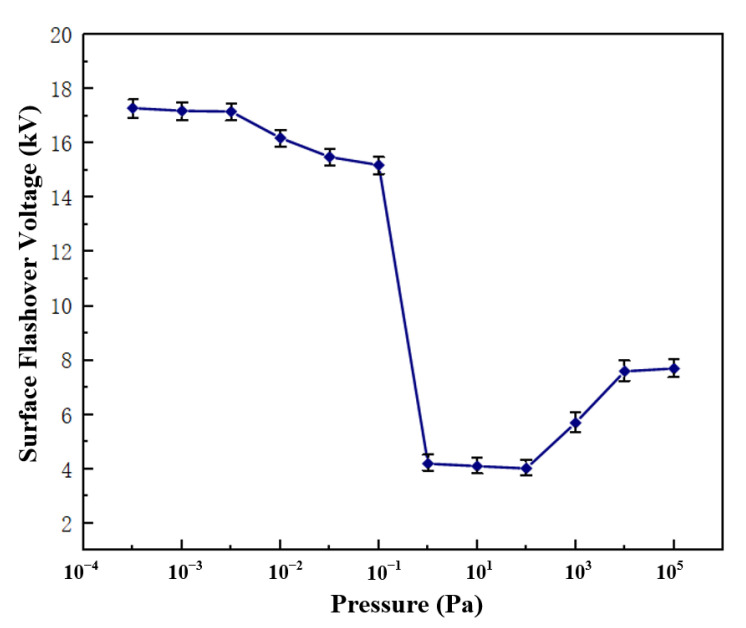
Surface flashover voltage of film under different air pressures.

**Figure 8:**
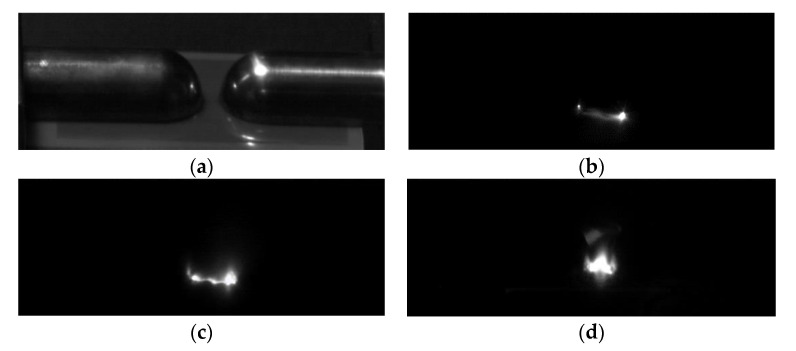
The flashover phenomenon along the surface of the film. (**a**) surface flashover platform; (**b**) flashover initiation; (**c**) form flashover channel; (**d**) surface flashover.

Using the PP5 sample with a POSS content of 5%, the surface flashover voltage of the composite material under different air pressures was tested, and the results are shown in Figure 7. The change of the flashover voltage along the surface of the material is divided into three stages: when the air pressure is less than 5 × 10^−3^ Pa, the flashover voltage tends to be stable and no longer changes with the change of air pressure; when the air pressure rises from 5 × 10^−3^ Pa to 10^2^ Pa, the flashover voltage showed a downward trend; when the air pressure increased from 10^2^ Pa to the standard atmospheric pressure, the flashover voltage gradually increased and became stable. According to the secondary electron emission avalanche model proposed by Anderson [25], the secondary electrons in the vacuum environment mainly come from the desorbed gas on the surface of the material, and the generation of the desorbed gas comes from the excitation of electrons, both of which have nothing to do with the air pressure. Therefore, when the material is in a high vacuum environment, changes in air pressure will not affect the flashover voltage. Considering the curve change trend of the flashover voltage in Figure 7, the subsequent flashover test of the polyimide composite film is set to be performed in a high vacuum environment with a pressure of 1 × 10^−3^ Pa. Figure 8 shows the surface flashover phenomenon of PP5 composite film when the vacuum degree is 1 × 10^−3^ Pa taken by a high-speed camera.

**Figure 9:**
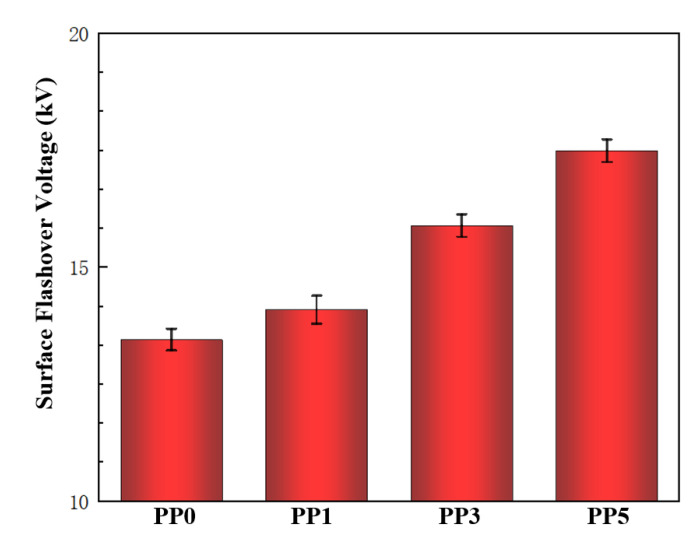
The flashover voltage along the surface of films.

The surface flashover experiments were carried out on four films with different POSS contents. Five experiments were carried out for each material and the average value of the surface flashover voltage was recorded. The results are shown in Figure 9. With the increase of POSS content, the flashover voltage along the surface of the composite films also increased gradually. Among them, the surface flashover voltage of the pure polyimide film without POSS is 13.4 kV; the surface flashover voltage of the composite film with 1% POSS content is 14.1 kV; the surface flashover voltage of the composite film with 3% POSS content is 15.9 kV; the surface flashover voltage of the composite film with 5% POSS content is 17.5 kV, which is 30.5% higher than that of the pure film.

**Figure 10:**
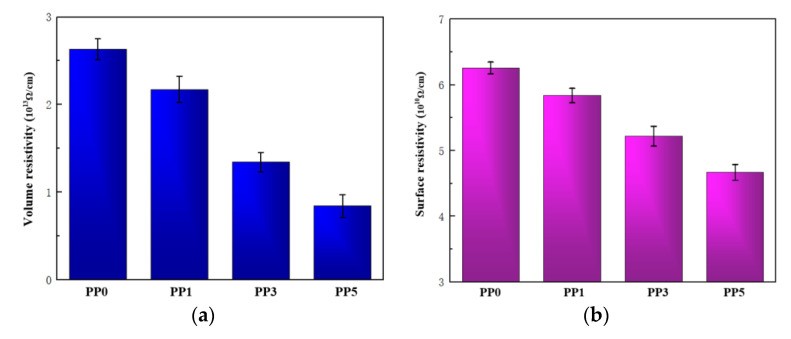
Resistivity of films. (**a**) Volume resistivity; (**b**) surface resistivity.

To reveal the mechanism of increasing the vacuum surface flashover voltage of polyimide films by introducing POSS units, the resistivity and surface microstructure of the films were studied. The development of material vacuum surface flashover will be affected by its resistivity, so the volume resistivity and surface resistivity of the film is tested, and the results are shown in Figure 10.

In the process of material surface flashover, the ability of the material surface to bind charges will have an important influence on the process. The surface charge not only distorts the electric field distribution on the surface of the material but also provides seed charges when the discharge develops, resulting in a drop in the flashover voltage [26]. Appropriate reduction of surface resistivity will weaken the surface charge accumulation ability of the material, which is beneficial to accelerate the surface charge dissipation speed. As shown in Figure 10, with the increase of POSS content, the volume resistivity and surface resistivity of the composite film gradually decreased. When the POSS content was 5%, the surface resistivity of the composite film was as low as three-quarters of the pure film. As the surface resistivity of the composite film decreases, the surface charge dissipation speed of the material also increases, and the electric field is more difficult to distort, thereby increasing the flashover voltage of the composite film. In addition, the reduction of the volume resistivity of the composite films is not simply affected by the POSS content but is related to the inter-aggregate distance [27]. When the POSS content is less than 5%, the aggregate volume density of the composite film increases with the increase of the POSS content, so the volume resistivity of the film decreases.

**Figure 11:**
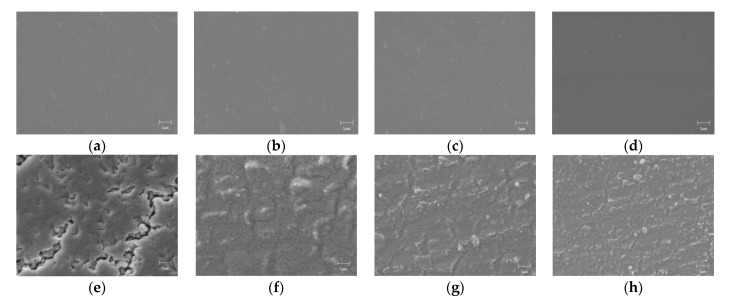
SEM micrographs of films. (**a**) PP0 before flashover; (**b**) PP1 before flashover; (**c**) PP3 before flashover; (**d**) PP5 before flashover; (**e**) PP0 after flashover; (**f**) PP1 after flashover; (**g**) PP3 after flashover; (**h**) PP5 after flashover.

The scanning electron microscope of films is shown in Figure 11: (a), (b), (c) and (d) are the surface morphologies of PP0, PP1, PP3, and PP5, respectively. It can be seen that the film after the introduction of POSS still maintains a good aggregation state. The surface of the film was smooth, the matrix was relatively uniform, and no phase separation was observed. (e), (f), (g), and (h) are the surface morphologies of PP0, PP1, PP3, and PP5 films after vacuum surface flashover, respectively. After the vacuum surface flashover occurred, the surface layer of PP0 was damaged in a large area, large groove-like dents were formed in some areas, and the inner layer matrix was exposed. At the same time, the cracks on the surface of the film make the gas on the surface of the material further overflow, which intensifies the emission of secondary electrons and reduces the flashover voltage. However, the surface morphology of PP5 film after vacuum surface discharge is still relatively complete, and flocculent particles are dispersed in many parts of the surface. During the vacuum surface flashover process, the corona will continue to erode the surface of the film, and at the same time, the surface of the film will expose the active material and react with the oxygen atoms released inside the material, causing the polyimide molecular chain to undergo a ring-opening reaction and form on the surface of the material, phase-separated inorganic silica, and under the high temperature generated by the discharge, the silicon-oxygen structure inside the PP5 film will undergo an oxidative cross-linking reaction, resulting in the appearance of SiO_x_ inorganic flocculent particles on the surface of the film. The inorganic structure can effectively scatter electrons, thereby suppressing charge injection and protecting the internal materials. In addition, the SiO_x_ on the surface of the film effectively shallows the depth of trap levels [28], so the charges stored in the shallow traps can more easily escape from the traps, resulting in faster surface charge dissipation and higher flashover voltage.

## 4. Conclusions

In this paper, the molecular structure of polyimide was modified by introducing 1%, 3%, and 5% molar content of POSS. The physicochemical properties and vacuum surface flashover characteristics of the composite films were tested. Conclusions are as follow:

(1) After the introduction of POSS, the overall functional group structure of polyimide remains unchanged. The charge transfer complexation between molecular chains is inhibited, resulting in a relatively weak intermolecular force. In addition, since the cage-like structure of POSS reduces the packing density of the material, the light transmittance of the film is improved. The introduction of POSS will enhance the thermal conductivity and thermal weight loss temperature of the composite film to a certain extent. The mechanical properties of the composite films are slightly reduced, but they can still meet the requirements of most application scenarios.
(2) After the introduction of POSS, with the increase of POSS content, the surface dielectric strength of the film is improved in vacuum. When the POSS content is 5%, the vacuum surface flashover voltage of the polyimide composite film increases to 17.5 kV, which is 30.5% higher than that of the pure polyimide film.
(3) The influence mechanism of POSS molecular structure-modified polyimide on the dielectric strength of the film under vacuum was preliminarily elucidated. First, the decrease of the resistivity of the composite film accelerates the dissipation speed of its surface charge, which increases the flashover voltage; second, the POSS forms a uniformly distributed Si-O-Si network by chemically keying the polyimide molecular chain modification. When the surface of the composite film is damaged, SiO_x_ inorganic flocculent particles will be generated, which can not only scatter electrons but also reduce the depth of trap levels to accelerate the dissipation of surface charges and increase the flashover voltage.

Our results show that the electrical insulating properties of the composites are ideally enhanced after molecular modification of polyimide using POSS. Therefore, POSS/polyimide composites are good candidates for insulating materials required for power devices operating in a vacuum environment. However, when the POSS content is elevated, the mechanical properties of the films are affected, which may limit their future practical applications. How to improve the electrical insulating properties of materials and simultaneously enhance its mechanical properties will be the main challenge for future research work. Our laboratory is also conducting related research on this.

## Figures and Tables

**Table 1:** Modification scheme.

Type of Film		Monomer Content		
		POSS (%)	ODA/mol	PMDA/mol
Pure PI	PP0	0	0.015	0.0153
Modified group	PP1	1	0.015	0.015453
	PP3	3	0.015	0.015773
	PP5	5	0.015	0.016105

**Table 2:** Thermal and mechanical properties of POSS–polyimide composite films.

Type of Film	Thermal Properties			Tensile Properties		
	T_5%_ (°C)	R_w750_ (%)	λ (W/m·k)	T_S_ (MPa)	T_M_ (GPa)	E_b_ (%)
PP0	532 ± 1.15	57.2 ± 0.05	0.236 ± 0.0015	136.5 ± 1.29	1.85 ± 0.005	65.2 ± 1.01
PP1	539 ± 1.35	57.4 ± 0.06	0.238 ± 0.0016	131.4 ± 1.38	1.84 ± 0.004	57.3 ± 1.08
PP3	546 ± 1.55	57.9 ± 0.04	0.246 ± 0.0016	120.3 ± 1.57	1.84 ± 0.004	46.7 ± 1.04
PP5	552 ± 1.45	58.4 ± 0.04	0.252 ± 0.0014	107.6 ± 1.51	1.83 ± 0.004	30.3 ± 1.03

## Data Availability

The data presented in this study are available on request from the corresponding author.
